# Reconstruction of whistling deformity using V-Y advancement flap after primary cleft lip repair

**DOI:** 10.1093/jscr/rjab026

**Published:** 2021-03-08

**Authors:** Sharon Claudia Notodihardjo, Kenji Kusumoto

**Affiliations:** Department of Plastic and Reconstructive Surgery, Kansai Medical University, Osaka, Japan; Department of Plastic and Reconstructive Surgery, Kansai Medical University, Osaka, Japan

## Abstract

Whistling deformity often occurs after cleft lip primary repair, marked by deficiency of vermilion tubercle. The purpose of the surgery includes improving the appearance and function of the lip. Adequate amount of vermillion tubercle is necessary to fully cover the front teeth, produce lip seal while speaking consonant sound and balance the projection of face.

Many techniques are known to repair whistling deformity from fat injection to Abbe flap. In our department, we use V-Y advancement flap. At certain cases, we repeat the procedure to maximize the result. This simple and less complication technique shows stable outcome during follow up. We recommend this technique because of the non-visible scar after surgery, applicable for unilateral and bilateral cleft lip case at various age, and possible to be done under general or local anesthesia.

## INTRODUCTION

Cleft lip palate is a major public health problem affecting one in every 500–1000 births worldwide. Asian populations have the highest frequencies, often at 1 out of 500 or higher. Prevalence rates of syndromic plus non-syndromic cleft lip with or without cleft palate among Japanese is 1.34 per 1000 live birth. Unilateral left side is dominant and followed by bilateral side [1, 2].

Patients with cleft lip and palate are usually operated on within the first year of life. The long period of dramatic growth and the resultant scar after the repair of the cleft structures, both profoundly affect the end result. Despite of any technique used for primary repair, the secondary deformities after cleft lip, cleft nose and palatal repair are the rule, not the exception. One of the secondary deformity that is common after bilateral cleft lip repair is deficiency of the midline vermilion, called ‘whistling deformity’ [3, 4].

Whistling deformity presents as notching or poor vermilion with exposure of central incisors in repose [5]. The lips’ surface can be divided into three portions: cutaneous, dry vermilion and wet vermilion. Underlying these superficial layers is a thin layer of connective tissue and fat, followed by muscles, which give the structural and functional of lip. Whistling deformity is not only unsightly, but may interfere with speech because of inability to produce lip seal, which is necessary to form the plosive consonant sounds [6]. The right surgical approach is important to improve esthetic and function. Identification of the deformity is important, whether it involve the skin only or also the muscle and mucosa layer. The methods vary from scar revision, local flaps, fat grafting or Abbe flap [7].

In current study, we showed series of our cases of whistling deformity reconstruction. We showed our surgical technique and outcome of the V-Y advancement flap.

**Figure 1:**
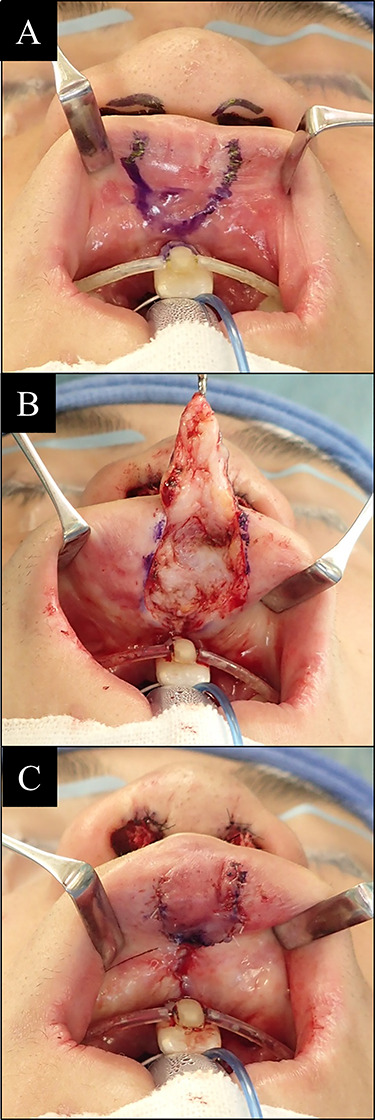
Reconstruction surgery steps of vermillion tubercle deformity. (**A**) Incision line design of lip mucosa; (**B**) flap was dissected until the muscle layer exposed; (**C**) flap then being advanced to more protrusive position and sutured.

**Figure 2:**
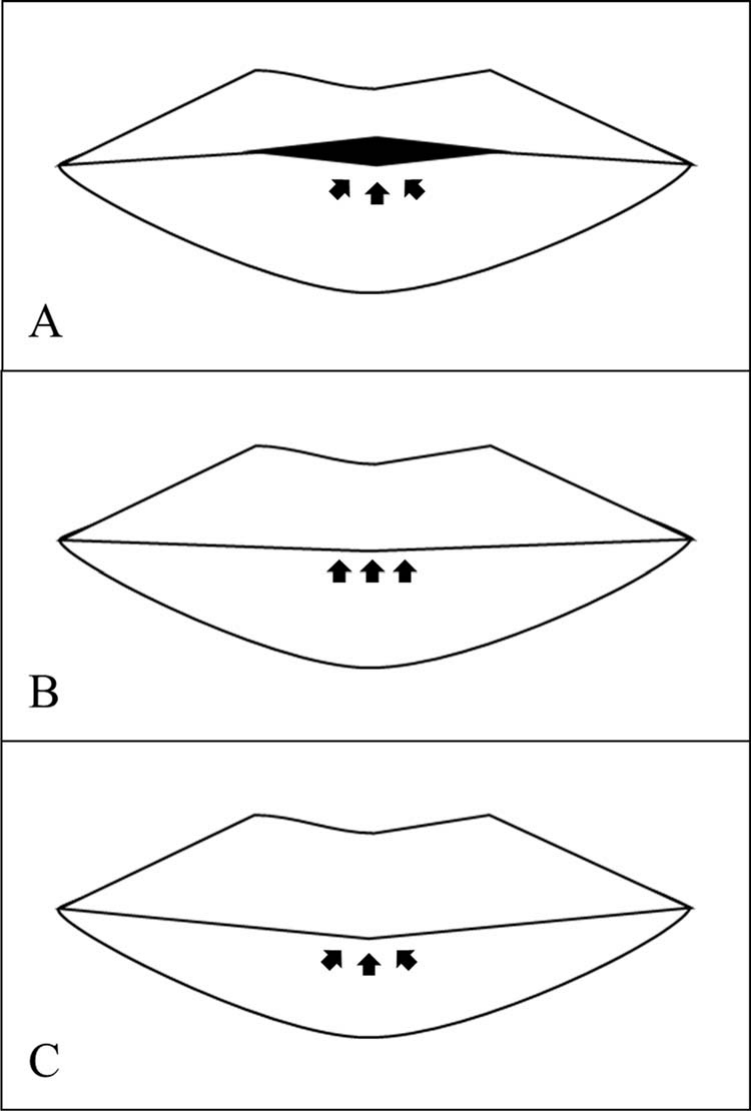
Three structure of lip vermilion tubercle. (**A**) Typical whistling deformity lip is deficit of vermilion tubercle, and incomplete lip seal is marked by arrows; (**B**) flat vermilion tubercle has complete lip seal, but the volume of tubercle is not full as normal; (**C**) ideal lip is marked by complete lip seal, enough volume of vermilion tubercle and balance of upper and lower lip.

**Figure 3:**
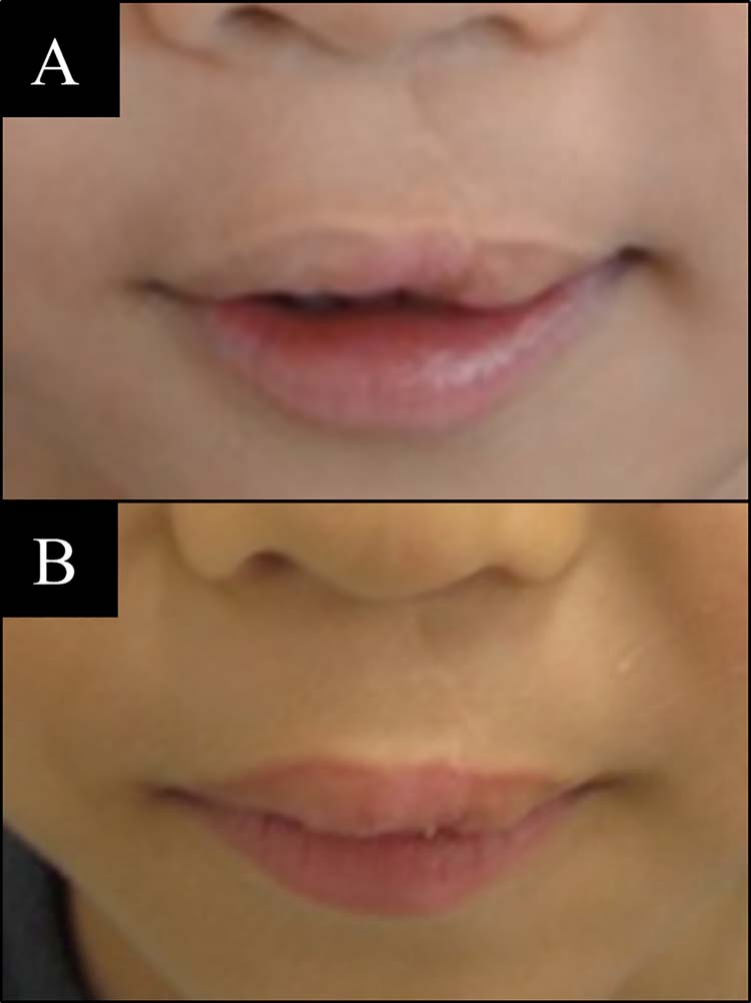
Case 1 with unilateral left side cleft lip. (**A**) Before vermilion reconstruction surgery and (**B**) 10 months after the V-Y advancement flap surgery.

## PATIENTS AND METHODS

All patients have signed informed consent for publication of their case details and accompanying images. All medical records were anonymous.

**Figure 4:**
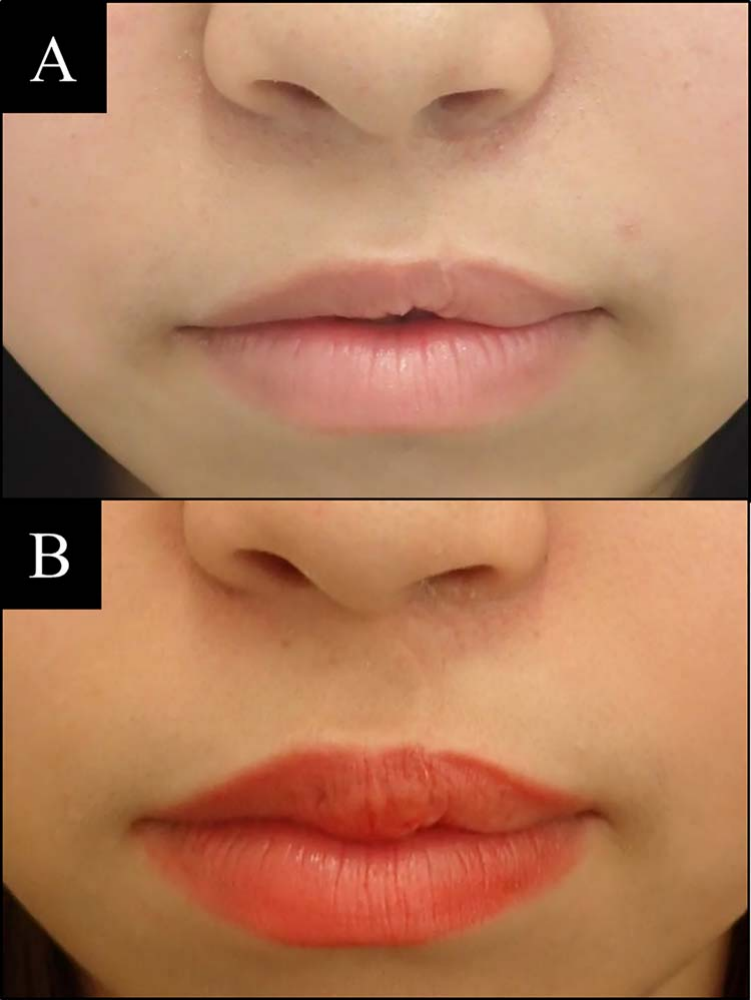
Case 2 with unilateral left side cleft lip. (**A**) Before vermilion reconstruction surgery and (**B**) 2 years after the V-Y advancement flap surgery.

**Figure 5:**
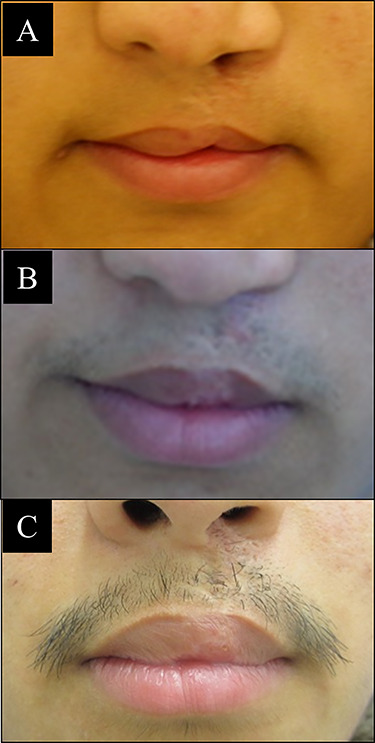
Case 3 with unilateral left side cleft lip. (**A**) Before vermilion reconstruction surgery; (**B**) 6 months after the first V-Y advancement flap surgery; (**C**) 2 years after the second V-Y advancement flap surgery.

### Clinical data

Between January 2006 and December 2019, we performed V-Y advancement flap at 49 patients post cleft lip surgery at our Plastic Surgery and Reconstructive Department. Among them, 29 patients were male and 20 patients were female. Surgery was performed at various age from 5 to 48 years old. Cleft lip is mostly unilateral left side with 22 patients, followed by 21 patients bilateral and six patients unilateral right side. The dominant characteristics are male gender (59.18%), went to surgery at age 12–18 years old (44.9%) with left side cleft lip (44.9%).

### Surgical technique

Surgery, as presented in Fig. 1, was performed either under local anesthesia or general anesthesia. V-shaped incision line design was made by skin marker at the upper lip vermilion defect area. Local anesthesia solution of two-times-diluted 0.5% lidocaine with epinephrine was injected for bleeding control. Incision was made by surgical blade no. 11. Lip mucosa was dissected until the plane between fat and muscle layer. The muscle layer of lip vermilion was exposed. Flap then sutured at advanced Y-shaped position with 5-0 polydioxanone thread to reform the vermilion tubercle.

### Aftercare

Repaired area was left open during follow up period. Patients were being prescribed with antibiotic and analgesic for 3 days. Patients were allowed to drink and eat normally right after local anesthesia surgery or after relieved from general anesthesia effect. Follow up visits were performed at 3 months after surgery and annually after that if there is no complication. At some patients, V-Y advancement flap surgery was performed two or three times to achieve maximum results.

## RESULTS

There is no complication such as flap necrosis or infection found after surgery. We compared the vermilion tubercle area before and after surgery, whether it is improved or not, as shown in Fig. 2. The best outcome is when the vermilion tubercle is nearly to the normal shape (Fig. 2C). The benefits of this V-Y advancement flap technique are applicable either for unilateral or bilateral case of cleft lip and at various age of patients. We can perform this surgery under general or local anesthesia. The scar after surgery is also hidden at the inner side of upper lip, which is important point at plastic surgery. We did repeated procedure of this technique at some patients, and there are no further complications. Some representative cases are shown below with different cleft side, age and number of surgery performed.

### Case 1

A 5-year-old male with left side cleft lip. A V-Y advancement flap surgery was done and followed up 10 months later. Result was stable and significant improvement can be seen (Fig. 3).

### Case 2

A 17-year-old female with left side cleft lip. The V-Y advancement flap surgery was performed one time and followed after surgery. The result is significant and stable after 2 years (Fig. 4).

### Case 3

A 15-year-old male with left side cleft lip. Three times of V-Y advancement flap surgery were done at the same defect site, with interval 2 years between each surgery. Desired volume of lip tubercle and balance between upper and lower lip was achieved and gave an excellent result (Fig. 5).

### Case 4

**Figure 6:**
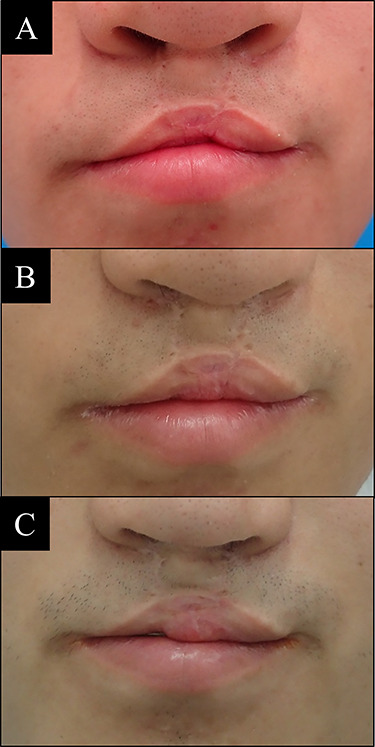
Case 4 with bilateral side cleft lip. (**A**) Before vermilion reconstruction surgery; (**B**) 1 year after the first V-Y advancement flap surgery; (**C**) 1 year after the second V-Y advancement flap surgery.

A 16-year-old male with bilateral side cleft lip. The V-Y advancement flap surgery was performed two times at a year interval. Final result has shown significant improvement compared with before surgery, but deficit of upper lip volume still exists (Fig. 6).

### Case 5

**Figure 7:**
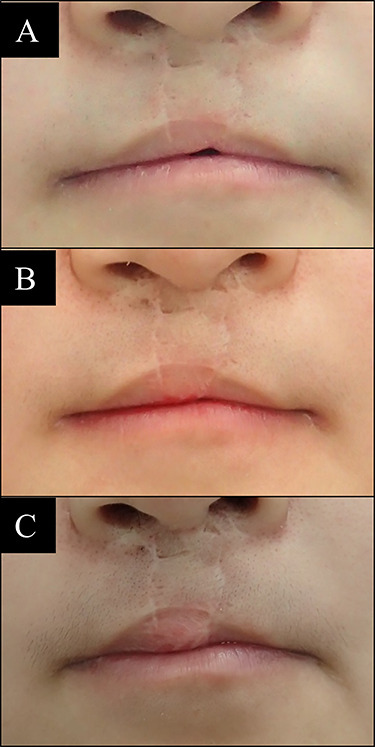
Case 5 with bilateral side cleft lip. (**A**) Before vermilion reconstruction surgery; (**B**) 8 months after the first V-Y advancement flap surgery; (**C**) 7 months after the second V-Y advancement flap surgery.

A 19-year-old female with bilateral side cleft lip. Two times of V-Y advancement flap were performed at 8 months interval. Final result has shown full covered incisor teeth compared with condition before surgery (Fig. 7).

## DISCUSSION

Lip has important esthetic features including the contour and volume, provided by soft-tissue bulk and configuration. The fullness at central vermilion is called as tubercle. At rest, the upper lip normally projects 2–3 mm anterior to the lower lip. With maximal smile, no more than ~1–2 mm gingiva should be exposed [4, 7].

After the primary repair surgery, patients with cleft lip will undergo at least one revision surgery when followed into adolescence, with a significant number of individuals requiring multiple interventions. The whistling deformity has been attributed to the sole use of mucosa from prolabial vermilion to form the central lip and the failure to attain continuity of the orbicularis oris muscle. This deformity can occur secondary to scar contracture across the vermilion, failure of mucosal restoration or failure to restore normal muscular continuity, as describe above [3, 8, 9].

If the central deficiency occurs with lateral excess, then advancement local flaps can be considered, such as V-Y advancement and Kapetansky’s pendulum flaps. If adequate amounts of vermilion are present and need augmentation, free fat grafting or other fillers material may be used. If the central tubercle vermilion bulk is deficient and there is no locally available tissue, using Abbe flap for correction is considered [3–5, 7, 10].

The V-Y advancement flap is a simple and less complication method to repair lip vermilion tubercle defect after cleft lip surgery. Compared with other method, incision field is relative small, which make it possible to be done under local anesthesia. We show averagely good result for both unilateral and bilateral side of cleft lip and no obvious scar in all our patients. This method is applicable for young and adult cases.

Despite of many modification techniques are established to repair vermilion tubercle, through this study we want to show that the classic V-Y advancement flap has significant benefit. This technique is very applicable and helpful especially for young plastic surgeon.

## CONFLICT OF INTEREST STATEMENT

None declared.

## FUNDING

None.

## References

[1] Cooper ME, Ratay JS, Marazita ML. Asian oral-facial cleft birth prevalence. Cleft Palate Craniofac J 2006;43:580–9. 16986997 10.1597/05-167

[2] Natsume N, Kawai T, Kohama G, Teshima T, Kochi S, Ohashi Y, et al. Incidence of cleft lip or palate in 303738 Japanese babies born between 1994 and 1995. Br J Oral Maxillofac Surg 2000;38:605–7. 11092775 10.1054/bjom.2000.0539

[3] Buchanan EP, Monson LA, Lee EI, Khechoyan DY, Koshy JC, Wison K, et al. Secondary deformities of the cleft lip, palate, and nose. In: Neligan P (ed). Plastic Surgery, 4th edn. London: Elsevier, 2018, 637–59.

[4] Stal S, Hollier LH. Secondary deformities of the cleft lip, nose, and palate. In: Mathes SJ, Hentz VR (eds). Plastic Surgery, 2nd edn. Philadelphia: Saunders Elsevier, 2006, 339–63.

[5] Harrison B. Cleft lip. In: Janis JE, Silverton JS (eds). Essentials of Plastic Surgery, 2nd edn. New York: Thieme, 2017, 264–74.

[6] Robinson DW, Ketchum LD, Masters FW. Double V-Y procedure for whistling deformity in repaired cleft lips. Plast Reconstr Surg 1970;46:241–4. 4915225 10.1097/00006534-197009000-00005

[7] Feldman EM, Koshy JC, Hollier LH, Stal S. Secondary deformities of the cleft lip, nose, and palate. In: Neligan PC, Rodriguez ED, Losee JE (eds). Plastic Surgery, 3rd edn. London: Elsevier/Saunders, 2013, 631–54.

[8] Millard R. Cleft Craft: The Evolution of Its Surgery—Volume II: Bilateral and Rare Deformities, 1st edn. Boston: Little Brown and Company, 1977, 453–68.

[9] Rodgers CM, Mulliken JB. Deepithelialized mucosal-submucosal flaps to correct the “whistling lip” deformity. Cleft Palate J 1989;26:136–40. 2706783

[10] Kapetansky DI. Double pendulum flaps for whistling deformities in bilateral cleft lips. Plast Reconstr Surg 1971;47:321–3. 4927274 10.1097/00006534-197104000-00003

